# Electrochemical corrosion resistance of aluminum alloy 6101 with cerium-based coatings in an alkaline environment

**DOI:** 10.3389/fchem.2022.1066958

**Published:** 2022-11-14

**Authors:** Ahsan Riaz Khan, Hai-Jun Zhang, Zhang Jun, Sayed M Eldin, Norah Saleem Alsaiari, Khadijah Mohammedsaleh Katubi

**Affiliations:** ^1^ Department of Interventional and Vascular Surgery, Shanghai Tenth People’s Hospital, Tongji University School of Medicine, Shanghai, China; ^2^ National United Engineering Laboratory for Biomedical Material Modification, Branden Industrial Park, Qihe Economic & Development Zone, Dezhou, Shandong, China; ^3^ Department of Chemical Engineering, Northwest University, Xi’an, China; ^4^ Research Center for Translational Medicine, Shanghai East Hospital, School of Medicine, Tongji University, Shanghai, China; ^5^ Shanghai Institute of Stem Cell Research and Clinical Translation, Shanghai, China; ^6^ Center of Research, Faculty of Engineering, Future University in Egypt, New Cairo, Egypt; ^7^ Department of Chemistry, College of Science, Princess Nourah bint Abdulrahman University, Riyadh, Saudi Arabia

**Keywords:** conversion, cerium, electrochemistry, coating, corrosion

## Abstract

Chromium-free materials as eco-friendly coatings with higher corrosion resistance are crucial in various industrial processes. Herein, we report the deposition of cerium-based conversion, a chromium-free, eco-friendly chemical conversion coating for aluminum alloy 6101, by the dip coating method. Immersion in cerium salt precursors assisted with hydrogen peroxide was performed for the deposition of cerium-based conversion coatings on aluminum alloy 6101 at different bathing temperatures. The electrochemical corrosion behavior was assessed in an alkaline solution of sodium hydroxide (pH 11), including mass loss measurements, free corrosion risk, polarization, and electrochemical impedance spectroscopy. X-ray diffraction and photoelectron spectroscopy analysis showed that the coatings were composed of Ce (III) and Ce (IV) oxides. Surface modifications and surface degradation of the coating and substrate after immersion in corrosive media were analyzed by scanning electron microscopy. Additionally, energy dispersive scanning analysis demonstrated the elemental composition before and after corrosion of the cerium salt conversion-based coating. The results demonstrated that selectively deposited cerium-based conversion coatings improved the corrosion resistance by up to 96% in a strong corrosive alkaline media.

## 1 Introduction

Conversion coatings are used to protect organic coatings from corrosion and to improve their adhesive properties. Conversion coatings have been used in industry for over a century. Conversion coating is a process that changes the surface metal oxide to a coating with different properties that integrates metal cations from the base metal (Li et al., 2022; Campestrini et al., 2004; Andreeva et al., 2016). The need to include cations originating from the substrate metal is removed by the broader definition. The industrial use of these coatings requires efficient processes (Castano et al., 2015; Zaman et al., 2021; Khan et al., 2022a). In recent years, the research community has adopted the phrase to represent any base-level rust coverage but does not always adhere to the exact processing requirements dictated by industrial processes, a fact that must be considered in the literature. Thus, the methods presented in the literature may not meet the requirements for industrial applications (Chen et al., 2021; Chauhan et al., 2022).

Anticorrosive treatments such as chromate conversion coatings (CCCs) have been employed for aluminum, tin, zinc, and steel. CCCs, primer, and paint systems are commonly used to protect aluminum alloy components from corrosion, in which the film coating plays a unique role in the defense mechanism (Zaman et al., 2022a; Sharif et al., 2023). However, despite the feature and benefits, due to the hazardous and carcinogenic nature of CCCs chemical dip treatment techniques when mixed with sodium dichromate solutions, there is an urgent need for updated processes (Chen et al., 2015; Eslami et al., 2017). Despite extensive investigation, no appropriate substitute treatment has yet been reported. The environmentally friendly substitutes to CCCs that have been examined as possible replacements include anodizing, rare-earth coatings, and pigments (Fahrenholtz et al., 2002; Harvey, 2013).

Due to their low costs, high strength-to-weight ratios, and corrosive resistance, aluminum alloys are widely used in various industries including automotive, aerospace, architecture, and desalination sectors (Khan et al., 2022c; Sharif et al., 2022). However, the different microstructures of aluminum alloys are vulnerable to local corrosion due to interactions between chloride ions (adsorbed on the substrate surface) and aluminum oxide layers, which reduces the metal thickness (Hughes et al., 2004; Khan et al., 2022c).

Rare-earth elements such as Nd, Pr, La, and Ce (Hasannejad et al., 2008; Zaman et al., 2022b) provide extraordinary resistance to localized corrosion by forming insoluble hydroxide/oxide layers. These elements are not considered hazardous due to their low toxicity. Therefore, coatings containing Ce and other rare-earth elements have been suggested as prospective substitutes for chromate-based preparations in metallic finish processes for aluminum alloys (Hughes et al., 2004; Joshi et al., 2012).

Hinton et al. (2003) presented one of the most researched systems, containing different rare-earth salts consisting of ions that produce unsolvable hydroxides with extraordinary resistance to localized corrosion (Kamde et al., 2021; Khast et al., 2022). The chemical reduction of (H_2_O_2_) causes the rapid deposition of Ce at cathodic sites in an aqueous media comprehending Ce (III) ions with H_2_O_2_ (Kulinich et al., 2007). The formation of hydroxyl ions in the cathode leads to a local increase in pH on the alloy surface, which promotes the formation of precipitates or/soluble ionic complexes in Ce(OH)_3_ such as ${Ce\left(OH\right)}_{2}^{\;2+}$ .

Generally, Ce (III) is thought to be oxidized to Ce (IV) in solution because it contains oxidizing agents (Kulinich et al., 2007; Kiyota et al., 2011). Since the Ksp of Ce(OH)_4_ is 4.010^−51^, the Ksp of Ce (OH)_3_ is significantly smaller (1.510^−20^). Ce (IV) deposition occurs at a lower pH compared to Ce(OH)_3_.

The present study evaluated the corrosion characteristics of Ce-conversion coatings on aluminum alloy 6101 based on the use of chloride and nitrate ions from Ce sources, conversion solution pH, different temperatures, and H_2_O_2_. The corrosive characteristics of alloy 6101 treated with the Ce-conversion coating, have not been thoroughly reported previously. Corrosion potential, polarization curve, XPS, and electrochemical impedance spectroscopy (EIS) have been used to investigate corrosion behavior supported by weight loss tests.

## 2 Material and methods

### 2.1 Materials

Specimens of commercially available aluminum alloy 6101 with nominal composition percents by weight of Si (0.3%–0.7%), Fe (≤0.50%), Cu (≤0.10%), Mn (≤0.03%), Mg (0.35%–0.8%), and Cr (≤0.03%) measuring 40 × 20 × 5 mm were tested. Analytical-grade chemicals with 99.99% purity, including cerium nitrate hexahydrate (Ce (NO_3_) _3_.6H_2_O) and hydrogen peroxide (H_2_O_2_), were obtained from the Tianjin Damao chemical reagent factory. Similarly, ethanol (CH_3_CH_2_OH), sodium hydroxide (NaOH), and nitric acid (HNO_3_) with 99% purities were obtained from Tianjin Fuyu Fine Chemicals Co., Ltd. (Table 1). Moreover, electro-coated and waterproof gelatin and carbide paper were obtained from Fuchen (Tianjin) Chemical Reagent Co., Ltd., and Matador Co., Ltd., respectively.

**TABLE 1:** Chemical compositions of Ce-based conversion coatings.

Conversion coatings	Concentration (mol L^−1^)	Composition of coating	Chemical formula
Coating	0.02 + 1 (30vol%)	Cerium nitrate hexahydrate + hydrogen peroxide	Ce (NO_3_) _3_.6H_2_O + H_2_O_2_

#### 2.1.1 Sample and surface preparation

Before the conversion coating treatment, the samples were mechanically polished to 1,200 g before being processed with silicon carbide (SiC) paper to enhance the surface modification for better adhesion of the Ce coating. To prevent surface defects, the following chemical pretreatments including an ethanol rinse and an acetone ultrasonic rinse for 10 min were performed at ambient temperature. Additionally, a 30 s cleaning, removal of dust particles present on the surface of the substrate with nitric acid (HNO_3_) solution, and etching in an alkaline solution of 1M sodium hydroxide (NaOH) were performed. After cleaning the surface of the aluminum alloy 6101 with acetone, the samples were rinsed with deionized water and stored in a desiccator for further experimentation to avoid surface defects and ensure the consistency of the surface hydroxides and oxides.

### 2.2 Coating formation and deposition

#### 2.2.1 Potential effects of temperature and heat on coating formation

**FIGURE 1:**
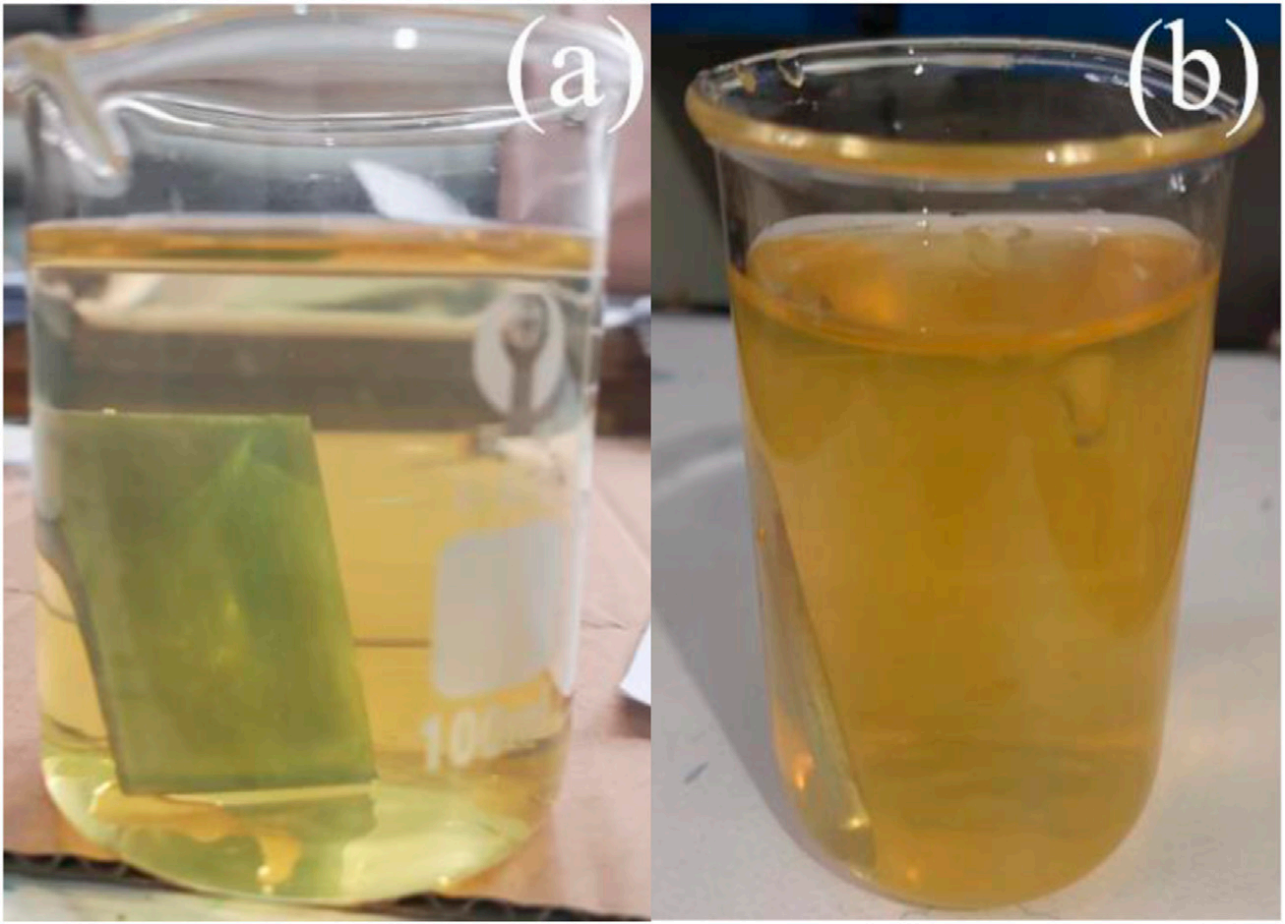
Two different colors of cerium conversion coatings at **(A)** 50°C and **(B)** 70°C temperature baths.

The solution color changes are shown in Figure 1. Upon the addition of H_2_O_2,_ the solution gradually turned golden yellow after boiling to 50°C. Continuous heating of the mixture at 70°C resulted in the removal of the dark orange suspended particles. This change may be due to the reaction that occurs after the solution.

$$H_{2}O_{2}+2_{e-}\to 2{OH}^{-}$$ (1)

$${4Ce}^{3+}+O_{2}+{4OH}^{-}+{2H}_{2}O\to {4Ce\left(OH\right)}_{2}^{\;2+}$$ (2)

$${Ce\left(OH\right)}_{2}^{\;2+}+2{OH}^{-}\to {Ce\left(OH\right)}_{4}\to {CeO}_{2}+{2H}_{2}O$$ (3)

**FIGURE 2:**
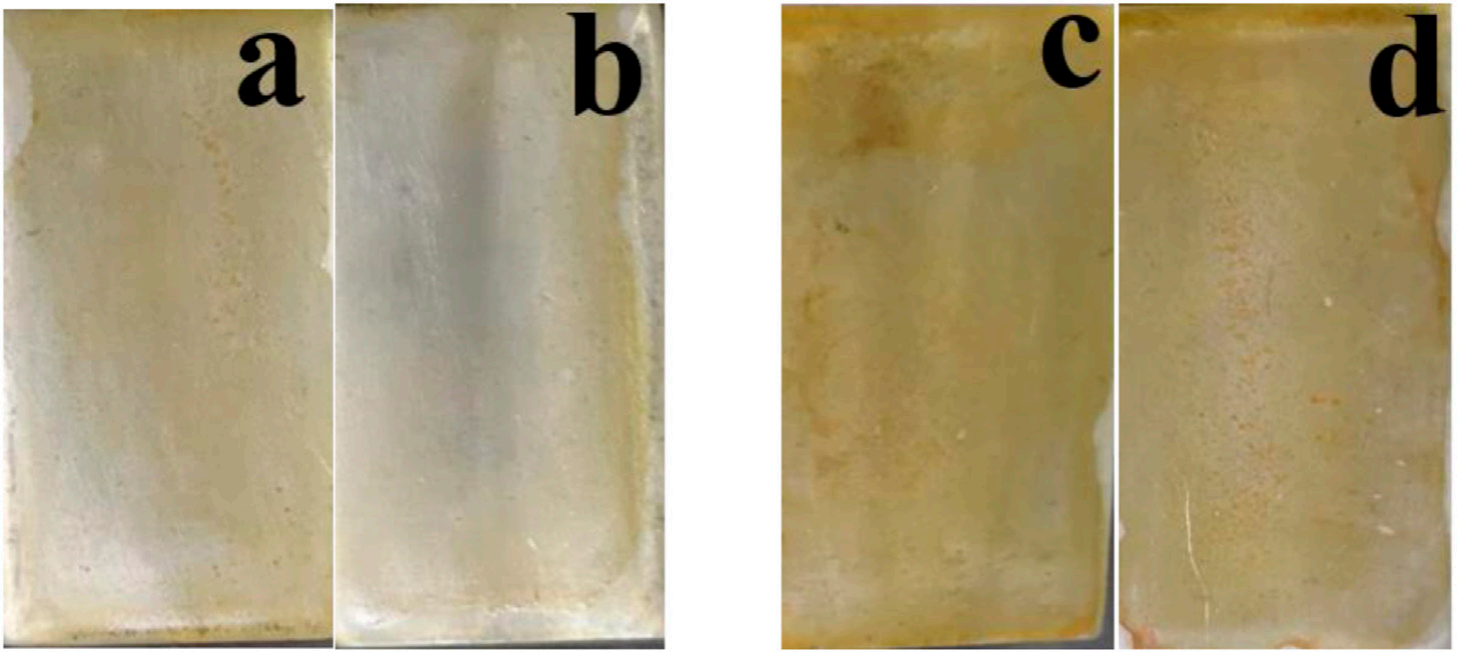
Coating deposition after the preparation of cerium conversion coatings. **(A,B)** At 50°C and **(C,D)** 70°C.

A high-temperature aqueous media along with a maximum consumption of H_2_O_2_ increases reaction kinetics, leading to the rapid utilization of coated-forming species in the cerium-based conversion media before the sample treatment. As the reduction of the number of reacting species also reduces the quantity of Ce coating the surface during the conversion coating treatment, the Ce–H_2_O_2_ transformation coating solution was first prepared at ambient temperature (25°C). The conversion coating was then applied in a solution continuously heated at 50°C–70°C (T70°C, 5°C/min heating rate for 8 min to stop the early fuming evaporation of the coating-forming species). Figure 2 shows the surface of the samples during coating at 50°C and 70°C. Under these temperature conditions, golden yellow surfaces formed, suggesting that cerium coatings formed under these conditions (Hasannejad et al., 2008; Chen et al., 2021).

#### 2.2.2 Coating deposition

**FIGURE 3:**
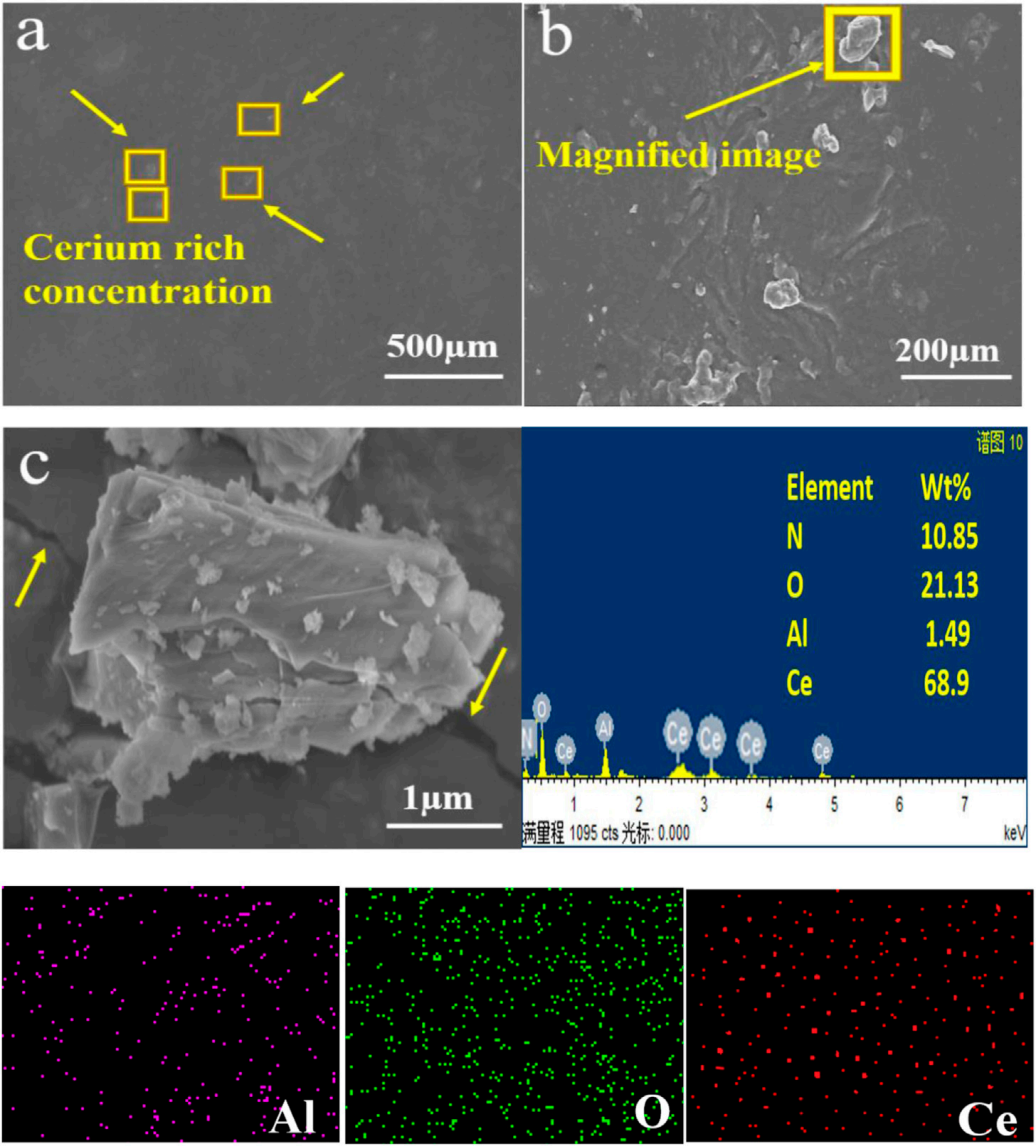
SEM images of the Ce-based conversion coating on the Al alloy 6101 before the corrosion test showing **(A)** homogenous coating on the substrate and **(B)** enrichment of the coating, as well as **(C)** EDS elemental analysis.

Different aqueous solution treatment temperatures were utilized as the sources of Ce for the chemical conversion coating. Solutions were produced at 50°C and 70°C, respectively, carefully agitated for 10 and 20 min, and utilized immediately after the addition of H_2_O_2_. A thermopotentiometer was used to measure the pH of the liquids (pH=5.2), which was adjusted by adding gelatin. Before the electrochemical experiments, the treated specimens were washed for 24 h at ambient temperature and dried. The chemical pre-treatment, coating temperature, reagent concentrations, and immersion time were pre-optimized in the laboratory. Single coatings are very thin and cannot resist the harsh corrosive environment for long; therefore, four rounds of coating were applied to the substrate to attain the desired coating thickness for better results (Figure 3). An average coating thickness of approximately 20 ± 5 µm was measured.

### 2.3 Surface morphology

The coating surfaces were observed by scanning electron microscopy (SEM). Energy-dispersion X-ray spectroscopy (EDS) and X-ray photoelectron spectroscopy (XPS) are used to analyze coating compositions (Khan et al., 2022a). The samples were PVD-plated with a Pt film before SEM and EDS analyses. X-ray diffraction (XRD) based on the 2500VB2+PC diffractometers in China was used to investigate the composition of the coatings using concentrated filtered Cu K radiation.

### 2.4 Corrosion test measurements

To characterize the corrosion resistance of the cerium-based conversion coatings, electrochemical and effective weight loss corrosion tests were performed. A PARSTAT 2273 potentiate electrochemical workstation was used for the electrochemical test measurements. The electrochemical station consists of a three-electrode (250 ml) cell, a working electrode (coated aluminum alloy), a platinum spade, and a saturated calomel electrode (SCE) with an auxiliary capillary as the reference electrode. The effective weight loss corrosion test was carried out by immersing the aluminum test sample in a basic solution of NaOH at pH 11 for 72 h, with weight readings every 12 h. The basic NaOH solution (pH 11) was used due to the severe aluminum alloy corrosion in alkaline conditions (Li et al., 2013; Mohammadloo et al., 2014).

If the initial weight is $W_{0}$ , after corrosion, the weight is defined as $W_{1}$ ; thus, the apparent corrosion rate due to weight loss is

$$V=\frac{W_{0}-W_{1}}{A\times t}$$ (4)

Where V is the apparent corrosion rate due to corrosion in mass change g/(m^2^⋅h), $W_{0}$ is the initial weight, $W_{1}$ is the final weight, A is the corrosion area, and t is the corrosion time.

An alternate form of Eq. 5 in depth is

$$V_{d}=\frac{8.75V}{\rho }$$ (5)

Where $V_{d}$ is the annual corrosion rate (mm/a) and ρ is the density of metal (g/cm^3^).

The protection efficiency can be calculated by following equation,

$$\eta \%=\left[\left(V_{dBlank}\;–\;V_{dCoated}\right)/\;V_{dBlank}\right]\times 100$$ (6)

where $C_{dBlank}$ and $C_{dCoated}$ represent the corrosion rates of the blank and coated metals, respectively.

## 3 Results and discussion

### 3.1 Mass loss corrosion test

After cleaning and drying, the effective weight losses of the bare and coated samples were measured to assess the apparent corrosion rates. The weight loss was measured by immersing the specimens in an alkaline solution of NaOH with a pH of 11 and measuring the weight change every 12 h. The alkaline solution was changed daily to maintain a constant pH. The specimens were then washed with deionized water before drying at 60°C for 20 min.

The samples with a cerium-based coating showed decreasing trends in weight loss and corrosion rates. Table 2 shows the mass loss and potentiodynamic polarization measurements at pH 11 for the coated and blank aluminum samples at various cerium salt concentrations. The mass loss showed an inhibition efficiency of 96.59% in the alkaline solution of NaOH with a coating efficiency of 52%. The inhibitor layer formed on the metal substrate protected the substrate surface from the corrosive media.

**TABLE 2:** Comparison of effective weight loss for Al alloy 6101 coated and blank samples in the NaOH corrosion solution (pH 11).

Serial no.	pH	V/g·m^−2^·h^−1^	Std. Dev., σ	V_d_/mm·a^−1^	η_w_%
24 h	11	0.90	0.24	0.016	96.59
48 h	11	1.06	0.18	0.020	92.77
72 h	11	6.68	0.22	0.017	52.91
Blank	11	14.3	-	0.265	-

### 3.2 Surface morphology

**FIGURE 4:**
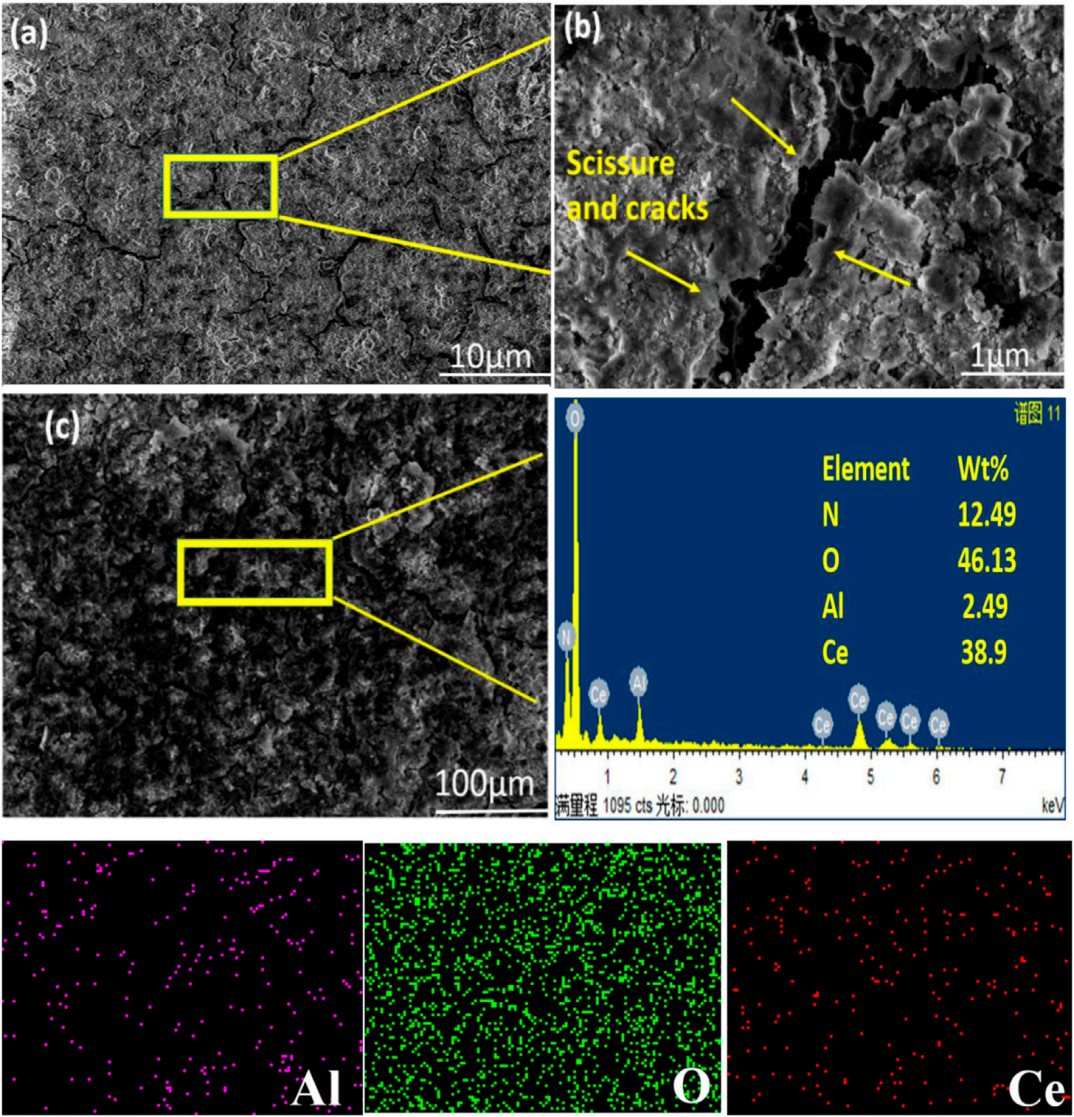
SEM images of the coating after 21 days of immersion in an alkaline NaOH solution. **(A)** showing cracks, **(B)** magnified mage, and **(C)** EDS of the elemental concentration.

SEM images were obtained of Al alloy 6101 with cerium oxide coating. Although the coating was stable and smooth, cracks were noted. Numerous researchers have cited the appearance of scissures and cracks typical of layers made from hydroxide due to the dehydration of cerium conversion coatings (Pardo et al., 2007; Paussa et al., 2012). An SEM image of the coating is shown in Figure 4, which comprises laminated layers and has a very fine thickness on the substrate surface. Numerous columnar formations were observed in each layer. Investigation of the conformation of the cerium-based eco-friendly coatings thru EDS and the elemental contents (wt%) showed N (12.49%), O (46.13%), Al (2.49%), Ce (38.09%). Therefore, the-dip coating of aluminum alloy 6101 cerium oxide-based mostly consisted of Ce, O, Al, and N elements (Sainis and Zanella, 2022).

### 3.3 XPS analysis

**FIGURE 5:**
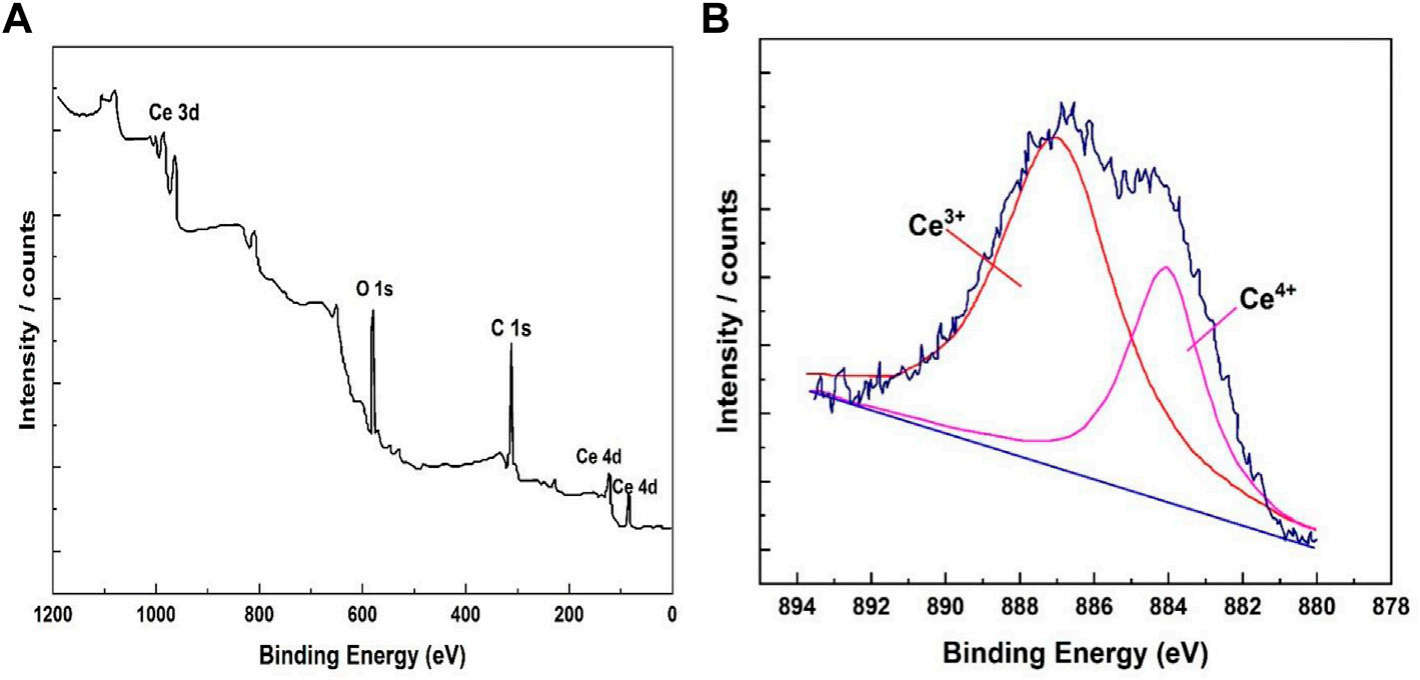
XPS band ranges of the cerium coatings. **(A)** Examination band spectrum. **(B)** Cerium-based conversion coating peaks.

XPS was used to assess the bonding of the elements in the coating. XPS analysis of the cerium-based coatings in Figure 5A showed the wide spectrum of Ce deposited on the coating surface. Ce, O, and C comprised most of the coating’s floor composition. The absence of Al peaks in the matrix showed that the coated surface was devoid of aluminum or its compounds. The overlapping Ce (IV) and Ce showed as the rare Ce peak between 878 and 892 eV (III). Similarly, Figure 5B shows the deconvolution of the Ce peak. The peak zone proportion of Ce (III)/Ce (IV), 0.45, was used to compute the comparative contents of cerium (III) and cerium (IV).

### 3.4 XRD characterization

**FIGURE 6:**
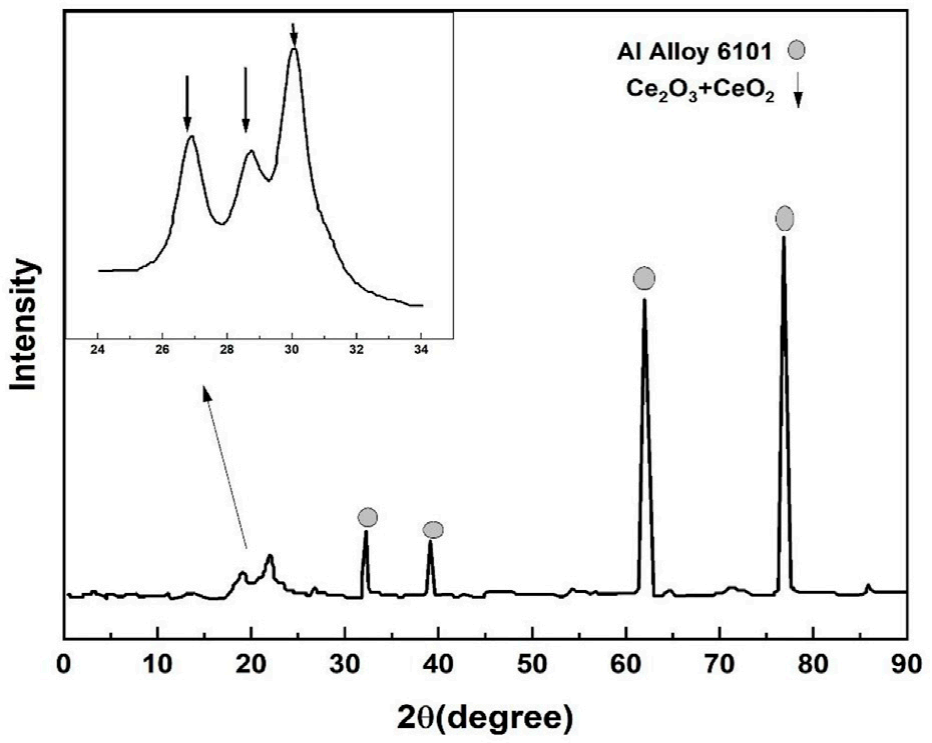
XRD peaks of the cerium coatings.

The XRD patterns of the dip-coated cerium conversion coatings are shown in Figure 6. The deflection peaks at 2θ = 25.417° and 2 = 28.445° could be related to planes (101) and (012) for Ce_2_O_3_ according to XRD. The small specific shoulder peak on the leftward side of the (002) peak may be due to the (111) replication of CeO_2_. The reason behind earlier reported cerium oxide coatings were primarily composed of Ce (IV) species, which may be related to the use of H_2_O_2_ in the conducting solution (Scholes et al., 2006).

The dip coating has been hypothesized to provide extra dissolved oxygen even without H_2_O_2_ in the coating solution. Throughout the dipping process, the coating species frequently settled in layers on the surface of the coated specimens (Tang et al., 2011), which may have caused more oxygen to dissolve in the electrolyte solution. Dissolved oxygen has less oxidizing power compared to H_2_O_2_. The oxidation of Ce (III) to Ce (IV) is difficult in single or spray coating processes, which may explain why the cerium conversion coating formed through dip coating was typically Ce (III) with Ce (IV).

### 3.5 Polarization curves

**FIGURE 7:**
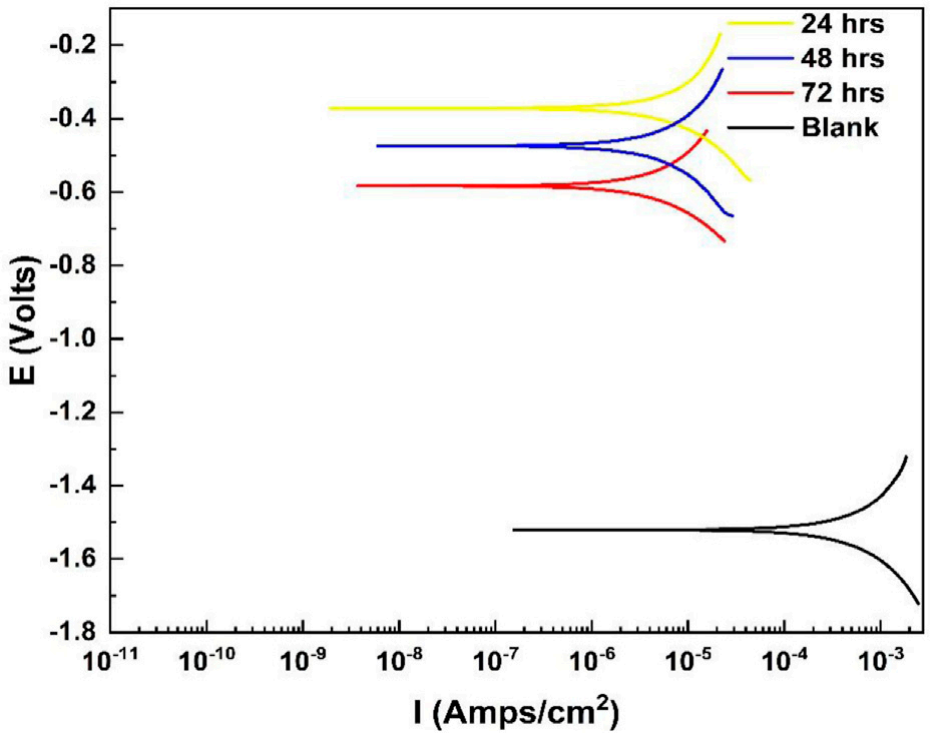
Tafel curves of the coated and bare Al alloy 6101 samples in the 0.001 M NaOH solution (pH 11).

Figure 7 shows the polarization curves for the untreated and treated specimens. The treated specimens showed higher positive electrochemical corrosion extracted values compared to those of the untreated specimens, showing that the Ce-oxide-based conversion coatings improved corrosion resistance by ennobling the potential and acting as anodic inhibitors. These findings are consistent with the electrochemical corrosive results. The enhancement in potential for specimens coated after treatment at temperatures of 50°C and 70°C was also assessed. Table 3 summarizes the Ecorr, polarization, and EIS data. The I_corr_ was calculated using polarization measurements and EIS data with the RP parameter. The I_corr_ value was significantly improved compared to that of the base material, which was reduced by approximately one order of magnitude. Both specimens had the lowest I-corr values, 0.4 or 0.6 L A cm^2^, respectively, which explained the observations of the coating degradation over time in the corrosive alkaline media, less intact, more absorbent, and less adhesive (Xingwen et al., 2001; Valdez et al., 2014).

**TABLE 3:** Comparison of electrochemical measurements for coated and blank Al alloy 6101 samples in the corrosion solution (pH 11).

Sample	pH	E_0_/V	I_0_/Amp/cm^2^ (E)	Ba/mV	Std. Dev., σ	Bc/mV	Corrosion rate (mm/a)	ηp,%
24 h	11	−0.624	1.254^–5^	28.17	1.80	−236.7	0.0090	98.59
48 h	11	−0.674	3.329^–5^	64.20	4.77	−285.9	0.0084	93.77
72 h	11	−0.583	5.329^–5^	66.00	5.26	−311.9	0.0550	52.91
Blank	11	−0.179	7.329^–5^	97.21	-	−138.9	-	-

### 3.6 Electrochemical impedance spectroscopy

**FIGURE 8:**
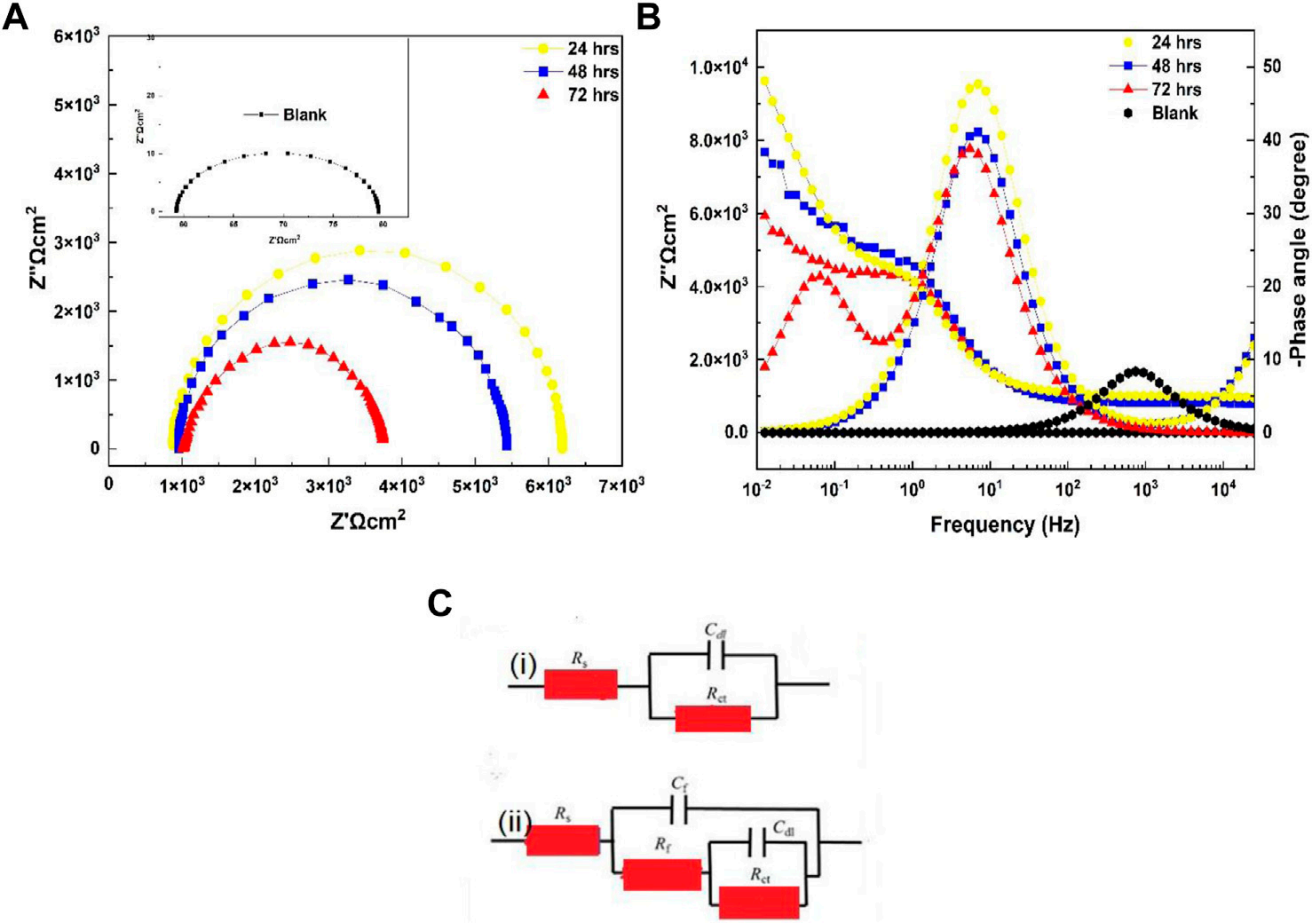
Diagram of electrochemical impedance spectroscopy. **(A)** Nyquist plot. **(B)** Bode and phase angle plots of blank aluminum 6101 with coated samples in a 0.001M NaOH solution. **(C)** Equivalent electrical circuits for EIS results: (i) blank aluminum and (ii) coated specimens.

At high-intermediate frequencies, the relaxation method exhibited a phase perspective (h) close to 45, indicating a capacitated behavior with outstanding di-electric characteristics; i.e., the conversion coatings could charge while avoiding the corroding solution’s ionic flux (Khan et al., 2022b). In contrast, the untreated 6101 specimen showed an excessive fee >70, indicating lower capacitive features compared to the treated specimens(Figure 8). Furthermore, the phase angle decreased with decreasing frequency, resulting in a second relaxation mode associated with penetration of the 0.001M NaOH solution into the underlying material surface *via* the coating’s pores.

Electrochemical analyses were performed under OCP conditions to measure the insulation behavior of the cerium-based coatings. The electrical equivalent circuit (EEC) utilized to characterize the Al alloy 6101 CeCC/NaOH machine significantly affected the interpretation of the EIS data. The fitting technique was carried out using EEC (Figure 8), which consisted of resistance (Rs) representing the ohmic electrolyte resistance, followed by the capacitance of the coating (Cf), another resistance (Rf) representing the coating properties, and a second parallel sub-circuit that associated the interface between the base material and the 0.001M NaOH solution across the porous grid of the coating, double-layer capacitance (Cdl) and charge transfer resistance (Rct) associated with the corrosion operation (Xingwen et al., 2001; Khan et al., 2022b), and a model used for the blank Al alloy 6010 sample showing resistance Rs for the naturally occurring oxide film present on the surface of the substrate, which was rapidly dissolved in the alkaline environment compared to the samples with cerium coatings. The arc-loop of the Nyquist plots decreased over time, with Rct values of 2.3 × 10^−6^ to 8.3 × 10^−4^ (Table 4), indicating a large coating adhesion loss in the hard alkaline-conducting solution. Moreover, the presence of the Ce oxide conversion coatings still protected the substrate surface and prevented the alkaline solution from infiltrating between the coating and the substrate surface.

**TABLE 4:** Electrochemical parameters from EIS equivalent electrical circuits of the specimens.

Parameters	Rs (Ω⋅cm^2^)	Cf (S⋅sn/cm^2^) (n)	Rf (Ω⋅cm^2^)	Cdl (S⋅sn/cm^2^) (n)	Rct (Ω⋅cm^2^)
24 h	971.1	1.86 × 10^–8^	6.729×10^5^	1.39 × 10^–5^ (0.574)	2.3 × 10^–6^
48 h	955.7	2.77 × 10^–8^	5.315×10^5^	1.53 × 10^–5^ (0.309)	1.6 × 10^–5^
72 h	908.7	3.57 × 10^–9^	4.566×10^4^	1.25 × 10^–5^ (0.354)	8.3 × 10^–4^
Blank	10.7	2.42 × 10^–9^	775.2	2.41 (0.282)	-

## Conclusion

Cerium-based conversion coatings were deposited on Al alloy 6101 after preparation by a dip-coating technique, which was performed four times to attain the desired coating thickness of approximately 25 ± 5 µm. These coatings were mainly cerium-based with good adhesive strength.

1) The coating chiefly contained cerium oxide and presented composite structures, which were ascribed to the repeated dipping process. The XPS results showed that the coating substrate on Al alloy 6101 was mainly protected by Ce (III) and Ce (IV).
2) The cerium-based conversion coating improved the corrosion resistance of the Al alloy 6101 by inhibiting the anodic and cathodic reaction rates in an alkaline environment.
3) Compared to the blank specimen, the cerium-conversion-coated Al alloy 6101 samples showed significantly better corrosion resistance in terms of I_corr_ and polarization resistance (Rp).
4) The eco-friendly conversion coating described in this study demonstrated its promise to replace hazardous chromate coatings. Finally, the conversion treatment significantly increased the corrosion resistance of the Al alloy 6101.

## Data Availability

The original contributions presented in the study are included in the article/Supplementary Materials. Further inquiries can be directed to the corresponding authors.
